# Severe multiple injuries in a 15-year-old boy with pelvic fracture complete anterior dislocation of the sacroiliac joint and rupture of the internal and external iliac arteries: A case report of a rare injury

**DOI:** 10.1097/MD.0000000000040015

**Published:** 2024-10-11

**Authors:** Yong-Gang Bao, Shu Li, Bao-Rui Liu, Yi-Feng Zhao, Fu-Qiang Song, Bin Wu

**Affiliations:** a Department of Clinical Medicine, Jining Medical University, Jining City, Shandong Province, China; b Department of Orthopedics, Affiliated Hospital of Jining Medical University, Jining City, Shandong Province, China.

**Keywords:** anterior dislocation of sacroiliac joint, multidisciplinary combined surgery, multiple trauma, pelvic fracture

## Abstract

**Rationale:**

Anterior dislocation of the sacroiliac joint combined with pelvic fractures is relatively rare in clinical practice. It is often associated with hemodynamic instability and severe injuries to other regions, resulting in a complex condition, prolonged treatment duration, and high rates of mortality and disability. However, there are few reports in the literature describing the diagnosis and treatment of anterior dislocation of the sacroiliac joint. In this case, the patient sustained a pelvic fracture with anterior sacroiliac joint dislocation and rupture of both the internal and external iliac arteries following a motor vehicle accident, making it an even rarer and more challenging case to treat. Reporting such cases can enhance the understanding of the diagnosis and treatment of anterior sacroiliac joint dislocation with rupture of the iliac arteries and provide valuable references for similar cases.

**Patient Concerns:**

The patient was riding an electric bicycle and was hit by a small truck, resulting in a pelvic fracture, anterior dislocation of the sacroiliac joint, and rupture of the internal and external iliac arteries.

**Diagnosis:**

The patient was diagnosed with open pelvic fracture (type C1.2), left complete anterior dislocation of the sacroiliac joint, left acetabular fracture, left internal and external iliac arteriovenous rupture.

**Interventions:**

emergency room resuscitation, intensive care unit resuscitation, 6 surgeries and perioperative management.

**Results:**

He has been discharged from the hospital for more than 1 year and was rechecked every month after discharge, the fracture has healed, there is no obvious pain and discomfort in and around the wound, he has been fitted with a prosthesis, and he is doing the walking function exercise.

**Lessons:**

Pelvic fracture with anterior sacroiliac dislocation is clinically rare and critical, and is associated with large vessel rupture, severe organ damage, and high mortality and disability rates. Rapid restoration of pelvic stability and hemodynamic stability is the key to treatment. Rapid transfer to a tertiary trauma center, rapid examination through the green channel to clarify the diagnosis, close intensive care, and reasonable multidisciplinary teamwork for surgical intervention are all valuable experiences that we have concluded.

## 1. Introduction

Obvious fracture dislocation of the sacroiliac joint is a subtype of complete pelvic fracture. Dislocation of the ilium anterior to the sacrum is frequently associated with joint separation and fracture of the pubic branch and ilium.^[1]^ Anterior sacroiliac dislocation is a rare injury-causing condition usually caused by violence or accidents, and few publications describe the diagnosis and treatment of anterior sacroiliac dislocation.^[2]^ The patient we treated had an anterior dislocation of the sacroiliac joint due to a pelvic fracture caused by a car accident injury combined with a rupture of the internal and external iliac arterial vessels, which is even rare and difficult to treat in clinical practice.

## 2. Case report

### 2.1. Current medical history

Patient Hou Mou, male, 15 years old, was admitted to the Affiliated Hospital of Jining Medical College as an emergency on May 29, 2022, for “post-traumatic generalized pain and bleeding for 5 hours.” Five hours before admission, the patient was hit by a car, and immediately after the injury, he felt pain in the chest, abdomen, left lower limb, and other parts of the body, restricted movement, accompanied by bleeding from the left groin and left knee, and coldness of the left lower limb, with no coma, impaired consciousness, headache, dizziness, chest tightness, palpitation, dyspnea, nausea, vomiting, and urinary and fecal incontinence.

### 2.2. Past history

The patient was in good health and had no history of hypertension, diabetes mellitus, coronary artery disease, or similar diseases.

### 2.3. Physical examination

**Figure 1.**
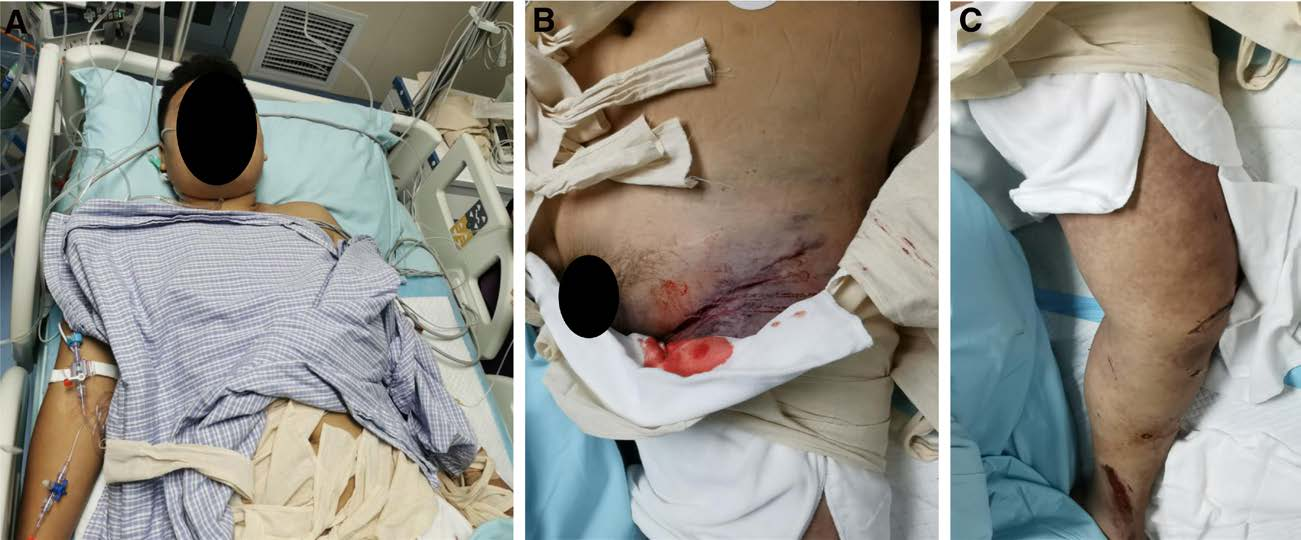
(A) Patient with anemic appearance after injury (B) Patient with bruising and swelling of the left ilium after injury (C) Patient with swelling and efflorescence of the left lower extremity after injury.

The patient was admitted to the hospital with vital signs: T: 36.3°C, P: 128 beats/minute, R: 21 beats/minute, BP: 108/59 mm Hg; physical examination: poor mental health, pallor (Fig. 1A), bruising of the left lower abdomen and pelvis, open wounds were visible, pelvic squeeze separation test was positive, large areas of bruising and swelling of the skin were visible on the left hip (Fig. 1B), multiple open wounds were visible on the left lower extremity, and the entire limb was swollen and obvious, bruising (Fig. 1C), deformity of the knee and ankle, decreased sensation of the entire left lower limb, impaired mobility, coldness of the limb, and dorsalis pedis artery and posterior tibial artery pulsations were not palpable.

### 2.4. Ancillary examinations

**Figure 2.**
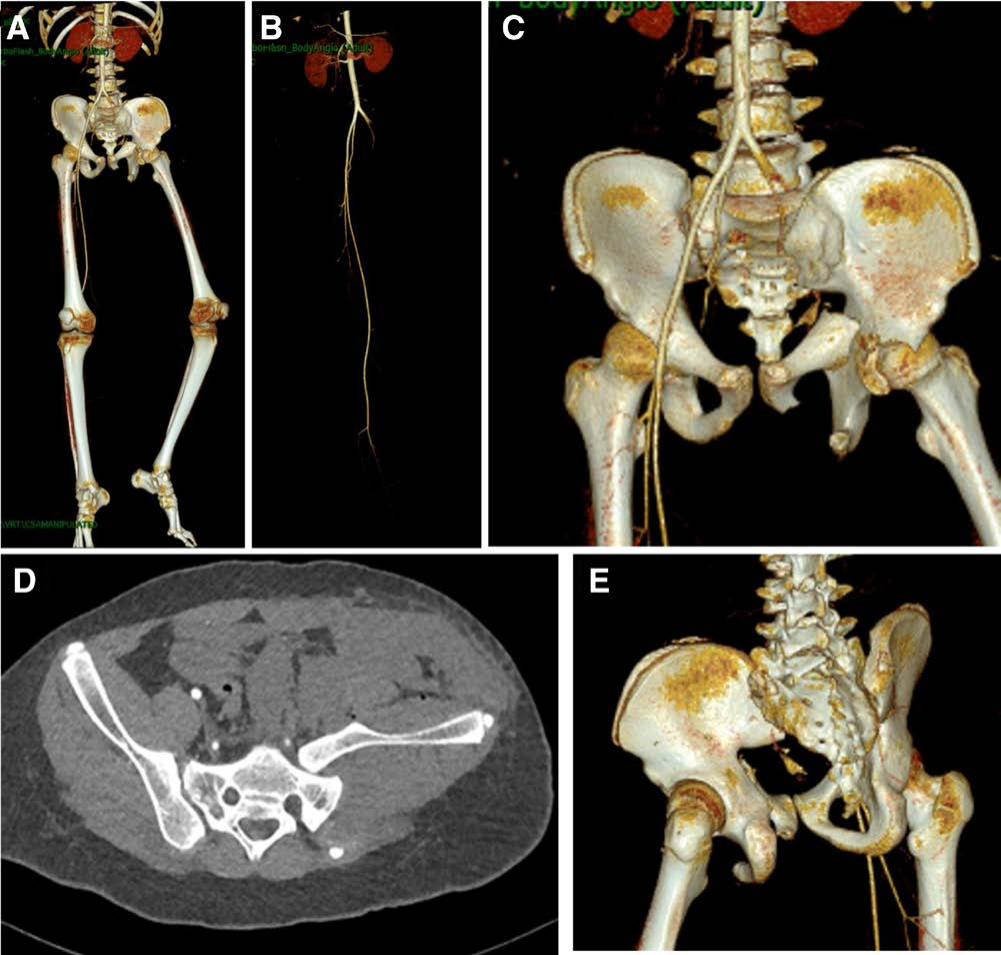
(A) Overall view of CTA 3D reconstructed image showing pelvic fracture (type C1.2), left complete anterior dislocation of the sacroiliac joint, and left acetabular fracture. (B) Angiographic shadow of CTA 3D reconstructed image showing left internal and external iliac arteriovenous dissection. (C) Anterior view of CTA 3D reconstructed image. (D) Cross section of CT image. (E) Posterior view of CTA 3D reconstructed image.

He was admitted to the emergency room and underwent blood tests with the following results: hemoglobin 67 g/L, prothrombin time 24.8 seconds, plasma fibrinogen 0.8 g/L. He underwent a CTA examination (Fig. 2), with the following results: fracture of the middle and distal portion of the left external iliac artery and the distal portion of the left internal iliac artery; anterior subluxation of the left sacroiliac joint; and fracture of the left acetabulum, the left upper and lower branches of the pubic bone, the left femur, and the left internal ankle.

### 2.5. Admission diagnosis

Traumatic hemorrhagic shock, multiple traumatic injuries, open pelvic fracture (type C1.2), left complete anterior dislocation of the sacroiliac joint, left acetabular fracture, left internal and external iliac arterial rupture, left pelvic crush and contusion, left lumbosacral nerve contusion, left lumbar nerve root avulsion, sacral nerve avulsion, closed chest, and abdominal injuries, hepatic and splenic contusions, abdominopelvic hematoma formation, left spermatic cord injury, left femur distal femur fracture, left medial ankle fracture, left lower extremity osteofascial compartment syndrome, and left lower abdominal skin dehiscence injury with effusion.

### 2.6. Treatment process

After the patient was admitted to the emergency room, the emergency room quickly initiated the process of severe trauma treatment, performed blood tests, and CTA examination, and was admitted to the monitoring room by green channel. The care unit continued to comprehensive treatment, due to the complexity of the patient’s condition, a single department is almost impossible to complete the rescue treatment. Therefore, the hospital immediately launched a multidisciplinary team (MDT) consultation mechanism, urgently convened experts from hepatobiliary surgery, urology, vascular surgery, and other related departments to fully discuss the case, and formulated a detailed diagnosis and treatment plan.

**Figure 3.**
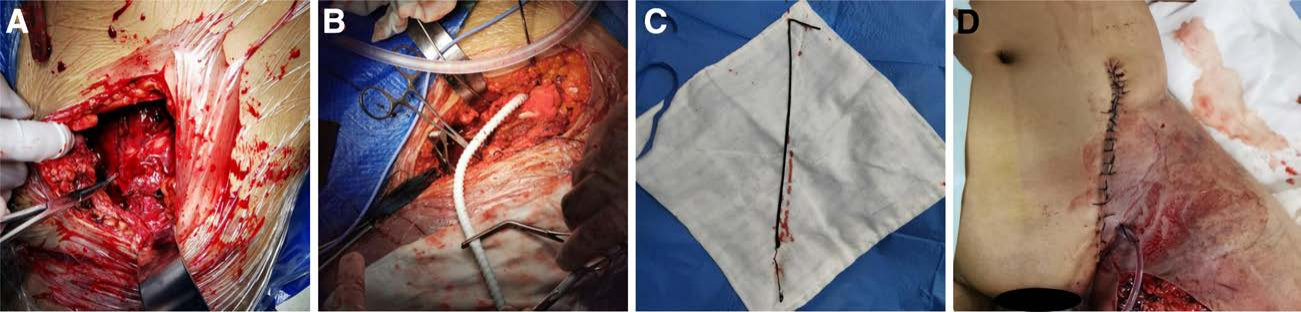
(A) Intraoperative auricular surface of the ilium. (B) Intraoperative artificial vessel bridging. (C) Intraoperative removal of thrombus in the distal artery. (D) Postoperative left hip incision left with negative pressure drainage of a coin, sutured layer by layer.

The multidisciplinary team agreed that only early combined multidisciplinary surgery could save the patient’s life and made the following plan: first, the medical, nursing, and administrative general duty were reported to coordinate the surgical team, surgical instruments, artificial blood vessels, and endopelvic fixation devices. In addition, the cardiopulmonary diversion team was notified to be in place to provide the necessary resuscitative measures. Subsequently, specialists from all departments were in place in the operating room. We then communicated with the family again before surgery. Finally, all specialists agreed that adequate preparations had been made to proceed with the surgery. After entering the operating room, pelvic fracture incision and reduction splint internal fixation, iliac artery-iliac artery artificial vascular bypass grafting (left) and limb osteofascial intercompartmental incision and decompression (left lower limb) were performed under static-aspiration compound anesthesia, and the operation was performed by trauma orthopedic surgeon, and the procedure was as follows: take the lying position after successful anesthesia, routinely disinfect the left lower limb, abdomen and perineum, and spread the sterile towel sheet. The left lower leg was swollen and externally rotated, and the abdominal wall was swollen and bruised. About 15 cm of lateral incision was made in the left lower abdomen, and the muscles of the external oblique, internal oblique, and transversus abdominis were cut layer by layer, and the deep bruise was obvious after separation, and the dark-red bloody fluid was attracted out of it (about 500 mL), and when the deeper part of the body was revealed, the iliac bone was externally rotated, and the anterior subluxation of the sacroiliac joints was seen, and it interlocked with the anterior aspect of the sacrum (Fig. 3A), and the upper and lower branches of the pubic bone were fractured. The upper and lower branches of the pubic bone were fractured anteriorly with obvious displacement, the anterior involved the anterior rim of the acetabulum, the external iliac artery was thwarted at the proximal end of the sacroiliac joint, the distal broken end was retracted back to the sub acetabular hair, thrombus was formed at the broken end, and the lumbar 5 nerve root and part of the sacral nerve were withdrawn, and completely dissected. First of all, clean up the bruise, clean up the fracture broken end, put Kirschner pin into the ilium, traction, prying and pivoting to reset the dislocated sacroiliac joint, 1 reconstruction plate, and 3 screws fixed, then reset the anterior suprapubic branch and the anterior wall of the acetabulum, Kirschner pin temporary fixation, 1 reconstruction plate and several screw fixation, the fracture reset and fixation of the lower extremity external rotation deformity corrected, and the mobility of the hip joint is good. The vascular surgeon assisted in the management of external iliac artery contusion, firstly, the proximal end of the vessel was treated, the stump of the vessel was trimmed to the normal vessel wall, flushed with heparin water, and an artificial vessel anastomosis was performed (Fig. 3B), with good blood circulation at 1 time, and then the distal end of the vessel was cleaned and trimmed to the normal vessel wall, and the embolus catheter was used to remove a dark-red thrombus of about 50 cm in length (Fig. 3C), and after seeing that the vessel was well circulated, the distal end of the anastomosis was anastomosed, and good blood circulation was achieved at 1 time. The external iliac vein was thwarted, and the severed end of the subsection was given to be ligated and no longer anastomosed, and the left hip incision was left with 1 negative pressure drainage and sutured layer by layer (Fig. 3D). After the anastomosis of the severed external iliac artery, the tension of the left thigh and calf was seen to increase gradually, the skin was tough and the tension was huge, and a high-pressure dissection and decompression of the fascial compartment of the left thigh and calf was given along the medial side of the left femur until the medial side of the left ankle, and the left thigh and calf muscles were seen to be dark red, with no obvious contraction on stimulation of the electrocautery knife, and the muscles were poorly activated; the inventory was correct, and there was no bleeding on probing activities and a thickened dressing was given to the trauma surface of the left buttocks and the left lower limb, and the trauma surface of the left hip and lower limbs was sent back to the ICU to stabilize He was sent back to ICU to stabilize his vital signs. Intraoperative bleeding was about 6000 mL, transfusion of B-type RH(D)-positive leukocyte suspension red blood cells 20 U, B-type virus inactivated plasma 800 mL, cold precipitation 12 U, the transfusion process was smooth, no transfusion reaction, transfusion of 5000 mL of fluids, urinary output 1500 mL.

**Figure 4.**
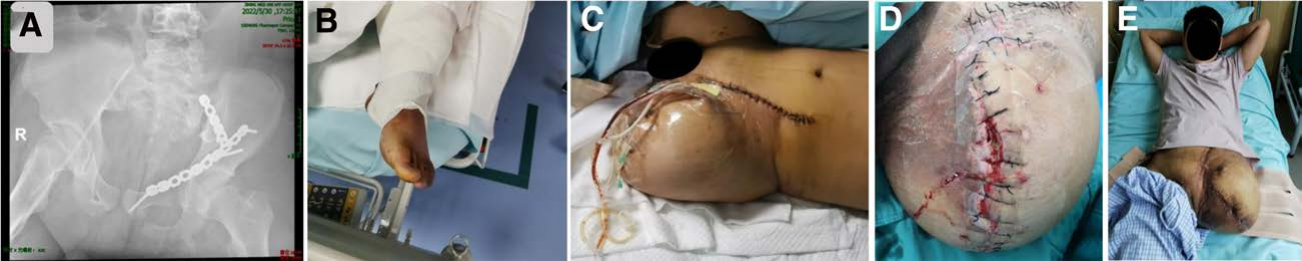
(A) Postoperative X-ray showing good alignment. (B) Poor postoperative peripheral blood flow. (C) After amputation. (D) Infection at the site of trauma after amputation. (E) Stump healing after amputation.

Postoperatively, the patient was transferred to the intensive care unit, where he was ventilated by endotracheal intubation and ventilator-assisted ventilation, and received close monitoring of vital signs. Postoperative X-rays showed good alignment (Fig. 4A), but 24 hours later, he developed left lower extremity florid and cold, poor peripheral blood flow (Fig. 4B), electrolyte disorders, acidosis, coagulation abnormalities, hypoproteinemia, and unstable vital signs. The patient’s left lower limb has ischemic necrosis, no possibility of limb preservation, if not amputation can be life-threatening, the family requested surgical amputation. Forty-eight hours after surgery, the left lower limb ischemic necrosis, and open amputation of the left thigh (Fig. 4C). The patient was in a coma for 4 days after the second surgery and was treated with continuous renal replacement therapy. The patient gradually woke up 7 days after the injury and developed a fever again, with a maximum temperature of 39.8°C. Infection of the trauma site appeared (Fig. 4D), and the bacterial culture was multidrug-resistant bacteria, 3 debridement surgeries were carried out, and the vital signs and all the inflammatory indices were gradually stabilized, but the stump trauma was still open. The stump was sutured for the next 40 days, and a small wound was left behind, which was treated with gradual dressing changes, and then the wound healed completely (Fig. 4E).

**Figure 5.**
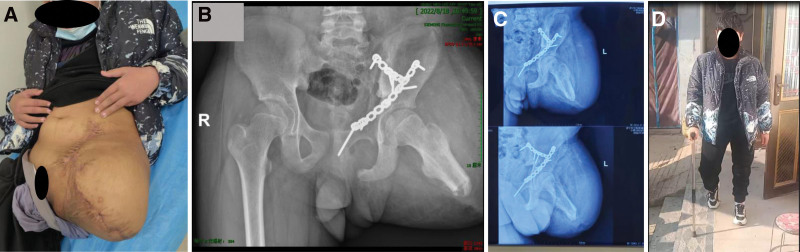
(A) The left lower abdominal and hip wounds were well healed at discharge. (B) X-rays at discharge showed a well-matched sacroiliac joint. (C) X-rays at 7 months postoperatively showed that the fracture had healed. (D) The patient was fitted with a prosthesis 1 year later and was doing functional exercises for walking.

After more than 2 months of 6 surgical treatments, multiple imaging examinations documenting the patient’s recovery, and a normal assessment of his condition, the patient was discharged from the hospital on July 2022. At the time of discharge, the patient’s abdominal trauma had recovered well: the trauma on the left side of the lower abdomen and the hip was healing well (Fig. 5A), with no redness, swelling, or exudation, and the bowel sounds were normal, with no abnormalities on perineal examination, and the bowel movements were normal; and the pelvic fracture had recovered well: a review X-ray showed (Fig. 5B), the left sacroiliac joint dislocation had been reset, anterior plate fixation, after internal fixation of the left suprapubic branch and the fracture of the anterior column of the acetabulum, the fracture had been healed, and the sacroiliac joint was well matched. Now he has been discharged from the hospital for more than 1 year, and he is rechecked every month after discharge (Fig. 5C), the fracture has healed, there is no obvious pain and discomfort in and around the wound, and he has been fitted with a prosthesis, and he is doing the walking function exercise (Fig. 5D).

## 3. Discussion

Pelvic fractures due to high-energy injuries are complex and serious, more than half of them are accompanied by comorbidities or multiple injuries, and the most serious ones are traumatic hemorrhagic shock and combined pelvic organ injuries, which are difficult to deal with clinically, and have high mortality rate when improperly rescued.^[3]^ In 1988, Tile redefined the Tile classification of pelvic fractures according to the stability of the fracture, the direction of the violence, and the nature of the fracture on the basis of Pennal classification, and classified the Pelvic ring fractures into type A, B, and C, which is the most commonly used classification.^[4]^ Significant fracture and dislocation of the sacroiliac joint is a subtype of complete pelvic fracture. Dislocation of the ilium anterior to the sacrum is frequently associated with joint separation and fractures of the pubic branch and ilium.

In this paper, the type of pelvic fracture was open pelvic fracture (C1.2), anterior sacroiliac joint dislocation with internal and external iliac artery, and vein rupture. Pelvic fracture combined with anterior dislocation of the sacroiliac joint is rare in clinical practice and is mostly reported in the form of case studies, with high morbidity, mortality, and disability, while pelvic fracture with anterior dislocation of the sacroiliac joint combined with dissection of the internal and external iliac arterial veins is even rarer, and is even more difficult to treat. Elnahal et al 2018 reported a pediatric patient with an anterior dislocation of the sacroiliac joint without a combined external iliac vascular injury, who received a good outcome using posterior sacroiliac joint fixation with an anterior external fixation frame, concluding that multidisciplinary collaboration and reconstruction of the stability of the pelvic ring is the key to a good outcome.^[5]^ Trikha et al reported 4 cases of anterior sacroiliac joint fracture dislocations and concluded that CT scanning is essential for diagnosis and preoperative planning of this injury pattern. Early repositioning of the iliac fragments helps in achieving anatomical repositioning and good functional outcomes in such patients.^[6]^

For the diagnosis of open pelvic fracture (C1.2), anterior sacroiliac joint dislocation with internal and external iliac artery and vein rupture, The speed, accuracy, and repeatability of modern CT scanners, and the close connection with trauma departments have led to the widespread use of CT angiography (CTA) when the limb is at risk of ischemia or a potential source of life-threatening bleeding.^[7]^ Therefore, we activated the green channel immediately after the patient was admitted to the hospital and completed the CTA examination in the shortest possible time, which provided us with a basis for understanding the patient’s condition and determining the surgical plan. From the results, the patient’s injuries were so severe that he had to undergo emergency surgery to save his life.

The key aspects of surgery are trauma control, restoration of pelvic stability, and hemodynamic stabilization. Vascular injury after pelvic rupture may lead to serious complications or even death.^[8]^ Therefore, we firstly traction and pry reset the dislocated sacroiliac joint, 1 reconstruction plate and 3 screws fixation, then reset the anterior suprapubic branch and the anterior wall of the acetabulum, 1 reconstruction plate and several screws fixation, and the lower limb external rotation deformity was corrected after fracture reset and fixation. Then the external iliac artery was treated and anastomosed with artificial blood vessels. The thrombus was removed by a thrombectomy catheter, and the stumps of the external iliac vein were ligated.

After 6 surgeries in more than 2 months, the patient successfully overcame postoperative infections, electrolyte disorders, acidosis, coagulation abnormalities, hypoproteinemia, and unstable vital signs. The patient has been discharged from the hospital for more than 1 year. After discharge, the patient was reexamined once a month, the fracture has been healed, and there is no obvious pain and discomfort in the wound and the surrounding area, he has been fitted with a prosthesis, and he is now doing functional exercise walking. However, in this case, the patient developed signs of infection following amputation, and the condition only stabilized after several debridement procedures, which is rare in our previous clinical experience. This suggests that a comprehensive preoperative assessment of the patient’s overall condition is crucial, and the prophylactic use of antibiotics prior to surgery should be considered.

In conclusion, the successful treatment of this patient provides a valuable reference for similar cases. Pelvic fractures with anterior sacroiliac dislocation are clinically rare and associated with severe conditions, often accompanied by major vascular rupture and significant organ damage, leading to high mortality and disability rates. Rapid restoration of pelvic stability and hemodynamic equilibrium is crucial for effective treatment. Immediate transfer to a level III trauma center, rapid diagnostic evaluation through fast-track protocols, close monitoring in the intensive care unit, comprehensive preoperative assessment, prophylactic antibiotic use, and coordinated multidisciplinary surgical intervention are key lessons learned from this case.

## Author contributions

**Formal analysis:** Bao-Rui Liu, Fu-Qiang Song, Yi-Feng Zhao, Bin Wu.

**Writing – original draft:** Yong-Gang Bao, Shu Li.

**Writing – review & editing:** Bin Wu.
